# Biomass Accumulation and Cell Wall Structure of Rice Plants Overexpressing a Dirigent-Jacalin of Sugarcane (*ShDJ*) Under Varying Conditions of Water Availability

**DOI:** 10.3389/fpls.2019.00065

**Published:** 2019-02-13

**Authors:** Larissa Mara Andrade, Rafael Fávero Peixoto-Junior, Rafael Vasconcelos Ribeiro, Paula Macedo Nóbile, Michael Santos Brito, Paulo Eduardo Ribeiro Marchiori, Samira Domingues Carlin, Alexandre Palma Boer Martins, Maria Helena S. Goldman, Juan Pablo Portilla Llerena, Caroline Fregonesi, Dilermando Perecin, João Felipe Carlos de Oliveira Nebó, Antonio Figueira, Thiago Romanos Benatti, Jorge da Silva, Paulo Mazzafera, Silvana Creste

**Affiliations:** ^1^Instituto Agronômico (IAC), Centro de Cana, Ribeirão Preto, Brazil; ^2^PPG - Genética, Faculdade de Medicina de Ribeirão Preto, Universidade de São Paulo, Ribeirão Preto, Brazil; ^3^Department of Plant Biology, Institute of Biology, University of Campinas, Campinas, Brazil; ^4^Instituto de Ciência e Tecnologia, Universidade Federal de São Paulo, São José dos Campos, Brazil; ^5^Instituto Agronômico (IAC), Centro de Ecofisiologia e Biofísica, Campinas, Brazil; ^6^Departamento de Biologia, Universidade Federal de Lavras, Lavras, Brazil; ^7^Departamento de Biologia, Faculdade de Filosofia, Ciências e Letras de Ribeirão Preto, Universidade de São Paulo, Ribeirão Preto, Brazil; ^8^Faculdade de Ciências Agrárias e Veterinárias, Universidade Estadual Paulista Júlio de Mesquita Filho (UNESP), Jaboticabal, Brazil; ^9^Centro de Energia Nuclear na Agricultura (CENA), University of São Paulo, Piracicaba, Brazil; ^10^Texas A&M Agrilife Research & Extension Center, Weslaco, TX, United States

**Keywords:** water deficit, RT-qPCR, transgenic plants, overexpression, monocot plants

## Abstract

A sugarcane gene encoding a *dirigent-jacalin*, *ShDJ*, was induced under drought stress. To elucidate its biological function, we integrated a *ShDJ*-overexpression construction into the rice Nipponbare genome via *Agrobacterium*-mediated transformation. Two transgenic lines with a single copy gene in T_0_ were selected and evaluated in both the T_1_ and T_4_ generations. Transgenic lines had drastically improved survival rate under water deficit conditions, at rates close to 100%, while WT did not survive. Besides, transgenic lines had improved biomass production and higher tillering under water deficit conditions compared with WT plants. Reduced pectin and hemicellulose contents were observed in transgenic lines compared with wild-type plants under both well-watered and water deficit conditions, whereas cellulose content was unchanged in line #17 and reduced in line #29 under conditions of low water availability. Changes in lignin content under water deficit were only observed in line #17. However, improvements in saccharification were found in both transgenic lines along with changes in the expression of *OsNTS1/2* and *OsMYB58/63* secondary cell wall biosynthesis genes. *ShDJ*-overexpression up-regulated the expression of the *OsbZIP23*, *OsGRAS23*, *OsP5CS*, and *OsLea3* genes in rice stems under well-watered conditions. Taken together, our data suggest that *ShDJ* has the potential for improving drought tolerance, plant biomass accumulation, and saccharification efficiency.

## Introduction

Sugarcane is a commercially important crop in tropical and sub-tropical regions (Inman-Bamber and Smith, 2005), and is the fifth most important crop in the world (FAOSTAT, 2013). Worldwide, sugarcane is considered the main raw material for table sugar production, and is being explored for the generation of clean and renewable energy, such as bioethanol and bioelectricity from first-generation ethanol (E1G) (Dias et al., 2011). In Brazil, the world leader in sugarcane production, the crop is cultivated over more than 9 million hectares, and the estimated production for the 2018/2019 season is around 365 million tons (CONAB, 2018).

In recent years, sugarcane production has been affected by unfavorable climatic conditions, which are increasing in frequency and intensity. Drought is an important abiotic stress that negatively impacts sugarcane productivity (Zhao and Li, 2015). This can be, due to water shortage even in rainy seasons or to the expansion of sugarcane cultivation to non-traditional planting regions, such as the Brazilian Cerrado (drought-prone conditions). Therefore, a challenge for sugarcane breeding programs is to develop cultivars with high productivity under water scarcity.

Despite advances in the conventional breeding of sugarcane, molecular biology and genetic engineering tools now have the potential to accelerate cultivar development and crop productivity by introducing new genes or manipulating gene expression. However, a lack of genetic and molecular information on drought tolerance mechanisms and their inheritance in sugarcane has limited the development of improved cultivars. Thus, functional genomics play a relevant role in the identification of target genes for the generation of transgenic sugarcane cultivars.

To withstand conditions imposed by water deprivation, plants have developed several strategies and responses on morphological, physiological, hormonal, molecular, and biochemical levels (Fang and Xiong, 2015). Among stress-responsive pathways, hormone signaling can regulate plant growth and enhance drought tolerance (Tiwari et al., 2017). Although abscisic acid is the principal mediator of drought responses, jasmonate (JA) plays an important role under abiotic and biotic stress (Wasternack, 2007), triggering response mechanisms that may improve stress tolerance (Muñoz-Espinoza et al., 2015).

The function of JA hormone signaling in the response to biotic stress is well-understood (Wasternack, 2007; Wasternack and Hause, 2013), and its involvement in the response to drought has been suggested (Muñoz-Espinoza et al., 2015; de Ollas and Dodd, 2016). JA promotes the activation of transcription factors resulting in the expression of various JA-responsive genes (Howe, 2010). The activation of JA-responsive genes can alter the levels of various proteins involved in numerous biological processes (Pauwels et al., 2009), such as lectin synthesis (Van Damme et al., 1998; Wang and Ma, 2005; Ma et al., 2010). Lectins are carbohydrate-binding proteins found in all organisms (Vijayan and Chandra, 1999; De Schutter and Van Damme, 2015), which recognize and reversibly bind to specific sugar structures and mediate several biological reactions (Peumans and Damme, 1995; Vijayan and Chandra, 1999). This heterogeneous group contains jacalin-related lectins (JRLs), which contain one or more JRL domains (De Schutter and Van Damme, 2015). JRLs have also been associated with an unrelated domain, and are named chimeric proteins (Jiang et al., 2010; Song et al., 2014). Several of these chimeric proteins (chimerolectins) contain domains related to stress response and defense. Of these, a C-terminal jacalin domain fused to a N-terminal dirigent (Song et al., 2014; Schutter and Van Damme, 2015) has been shown to affect a broad range of physiological functions in monocot plants (Ma, 2014; Song et al., 2014). Nobile et al. (2017) made a comprehensive characterization of proteins containing dirigent (DIR) domain in sugarcane and found 6.7% as chimeric jacalins containing DIR domains.

Monocot chimeric jacalins have been identified in maize (Esen and Blanchard, 2000), sorghum (Kittur et al., 2010), rice (Jiang et al., 2006, 2007; Hensel et al., 2016), wheat (Subramanyam et al., 2008; Ma et al., 2013), and sugarcane (Nobile et al., 2017). These proteins play important roles in both biotic and abiotic stress responses (Ma, 2014), and in the regulation of plant growth and development (Lannoo and Van Damme, 2010). In sugarcane, the exact functions of the JRL domain associated with a dirigent domain (Dirigent-Jacalin or DJ) have not yet been characterized. Despite the importance of plant JRLs, current knowledge on genome function and the regulation of JRLs in polyploid species, as in sugarcane (*Saccharum* spp.), remains elusive. Although several studies have addressed drought tolerance in genetically modified sugarcane (Zhang et al., 2006; Molinari et al., 2007; Kumar et al., 2014; Reis et al., 2014), the lack of well-characterized genes that guarantee satisfactory yield under water deficit conditions represents a bottleneck for the commercial generation of transgenic cultivars. Thus, elucidating the molecular mechanism that underlies drought tolerance in sugarcane is mandatory for developing new cultivars with improved drought tolerance. Therefore, our group has dedicated efforts to understand the molecular basis of drought tolerance in sugarcane aiming to identify candidate target genes to improve sugarcane yield under conditions of low water availability. In a previous experiment (Oliveira, 2012), transcriptome analyses (microarray and RNA-seq) of two sugarcane genotypes contrasting in drought tolerance were performed to identify stress responsive genes.

Among several candidate genes, *ShDJ* was upregulated in response to drought. Therefore, we chose the *ShDJ* gene characterized its role in drought tolerance. The *ShDJ* full-length coding DNA sequence (CDS) was cloned and overexpressed in rice, a monocot model used in functional genomics (Tyagi and Mohanty, 2000). We investigated the performance of transgenic rice lines constitutively expressing the *ShDJ* gene under varying levels of water availability. Our results indicated that constitutive expression of the *ShDJ* gene improved drought tolerance in transgenic rice lines and exerted a positive impact on biomass accumulation, an important trait for agriculture. Then, we further investigated possible changes in cell wall components (cellulose, hemicellulose, and pectin) and lignin composition, as these elements affect the production of lignocellulosic bioethanol (Bottcher et al., 2013), also known as second-generation ethanol (E2G). Biochemical analyses revealed that *ShDJ*-overexpression modulated pectin and hemicellulose components, and improved saccharification efficiency. Together, our findings may represent a disruptive technology for the development of a sugarcane cultivar overexpressing *ShDJ*, which would be drought tolerant and show higher biomass production with enhanced saccharification for the sugar, E1G and E2G industries.

## Materials and Methods

### Sugarcane *Dirigent-Jacalin* Gene Identification and Expression Analyses

In order to understand the mechanisms involved in the drought response of sugarcane plants, ‘IACSP94-2094’ (drought-tolerant) and ‘IACSP97-7065’ (drought-sensitive) sugarcane (*Saccharum* spp.) genotypes developed by “Programa Cana” (Instituto Agronômico, Ribeirão Preto, Brazil) were previously evaluated under irrigated and non-irrigated conditions both on field and greenhouse conditions (Oliveira, 2012). The field trial was carried out in Goianésia, Brazil (15°13′ S; 48°56′ W) during the dry season. Briefly, leaf samples (leaf +1) of first-cut plants were collected between 9:00 and 9:30 a.m. in irrigated (the irrigation was applied by linear sprinkler system) and non-irrigated areas at 42, 89, and 117 days after the last rainfall, when plants were 6, 7, and 9 months old respectively. The greenhouse trial was carried out in Campinas, Brazil (22°52′ S; 47°44′ W), and both genotypes were grown in the same tanks (0.6 m^3^) containing soil previously fertilized according to Van Raij et al. (1996). Leaf samples (leaf +1) from 6 months old plants were collected between 9:00 and 9:30 a.m. in irrigated and non-irrigated treatments at: 15 and 21 days after water withholding deficit and also after 9 days of soil rehydration for evaluating plant recovery. For more details about field and greenhouse trials, refer to Andrade et al. (2016). Leaf samples from both field and greenhouse experiments were subjected to microarray and RNA-seq assays, respectively (Oliveira, 2012). From these expression global analyses, *ShDJ* was chosen to be validated by real time quantitative polymerase chain reaction (RT-qPCR) in the present study.

Total RNA was extracted from leaves, according to Chang et al. (1993). Genomic DNA was removed using DNase I, following the manufacturer’s instructions (Promega, Fitchburg, WI, United States). RNA concentration was determined using a spectrophotometer NanoDrop 2000 (Thermo Fisher Scientific, Wilmington, DE, United States), and RNA integrity was checked in 1.0% agarose gel electrophoresis stained with ethidium bromide (1 μg mL^−1^). Reverse transcription reaction was synthesized from 1 μg of total RNA using the QuantiTect^®^Reverse Transcription Kit following the manufacturer’s instructions (Qiagen, Foster City, CA, United States).

Real time quantitative polymerase chain reaction reactions were performed on the Applied Biosystems StepOnePlus System (Foster City, CA, United States). Briefly, a 10 μL reaction mixture consisted of 5 μL SYBR Green Super Mix (Applied Biosystems, Foster City, CA, United States), 3 μL of diluted cDNA (1:30) with 0.2 μM primers concentration, besides a negative control (without cDNA) included for each primer combinations. Expression was evaluated by the 2^−ΔCt^ method [*n* = 3 ± standard error (SE)], which represents the relative quantification of ShDJ expression in relation to the UBQ1 reference gene (Andrade et al., 2017), as shown in Supplementary Table 1.

### Alignments and Phylogenetic Analyses

The *ShDJ* gene sequence (SUCEST Accession No. SCJLLR1103A10) was used as a bait for identifying its homologous using Basic Local Alignments Search Tools (Altschul et al., 1997) in different databases, such as the SUCEST database^1^, GenBank (NCBI^2^), and Phytozome^3^ (Supplementary Table 2). Sugarcane assembled sequences (SAS) were retrieved using the tblastn tool with a cut-off of E-value 2e^−56^, and a minimum SAS coverage rate in relation to the original protein sequence used as bait of at least 50%. Homologous *ShDJ* protein sequences obtained from sugarcane hybrid (Sh), *Sorghum bicolor* (Sb), maize (Zm), rice (Os), *Hordeum vulgare* (Hv)*, Brachypodium distachyon* (Bd), *Triticum aestivum* (Ta), and *Arabidopsis thaliana* (At) (Supplementary Table 2) were aligned the domain DJ proteins using ClustalW program (Thompson et al., 1994) based on Jaccard’s index of similarity. Phylogenetic analyses was generated and visualized using Mega 6 (Tamura et al., 2013), with the maximum likelihood cluster analyses based on the JTT amino acid substitution matrix (Jones et al., 1992). Rates among sites were obtained using Gamma Distributed (with five discrete gamma categories). Trees were generated using BIONJ (Gascuel, 1997), a modified neighbor-joining algorithm, and each node was tested with 1,000 bootstrap replicates.

### Construction of the *ShDJ* Expression Cassette and Rice Transformation

To construct the overexpression vector, the complete open reading frame (ORF) of *ShDJ* cDNA sequence was obtained using the SMARTer RACE cDNA Amplification Kit (Clontech, Mountain View, CA, United States), and the CDS was cloned into a pGEM-Teasy (Promega, Fitchburg, WI, United States). The binary vector pHb7m24GW, from the Functional Genomics unit of the Department of Plant Systems Biology (VIB-Ghent University), carrying the maize *ubiquitin* promoter (pEN-L4UBIL-R1) driving *ShDJ* expression, and the hygromycin phosphotransferase gene as a selectable marker (Karimi et al., 2007), was obtained by multi-recombination using the Gateway Recombination System^TM^ (Invitrogen Life Technologies, United States), and transferred to *Agrobacterium tumefaciens* strain EHA105.

Embryogenic *calli* were obtained from mature seeds of Japonica rice (*Oryza sativa* L. ‘Nipponbare’), and transgenic lines were produced as described by Toki et al. (2006), with modifications. Plants were regenerated on medium containing 30 mg L^−1^ hygromycin for selection and 20 mg L^−1^ Meropenem to prevent overgrow of *A. tumefaciens*. The progenies were obtained by self-pollination following the selection of seeds through a germination test on hygromycin-containing media. Seeds of *ShDJ* lines were screened on ½ MS medium supplemented with 50 mg L^−1^ hygromycin (incubation at 27°C for 7 days under a 16 h photoperiod) to obtain T_1_ and successive progenies for further analyses.

### Gene Expression Analyses of *ShDJ* Transgenic Lines

Total RNA was isolated from rice tissues as described by Chang et al. (1993). To nullify any genomic DNA contamination, isolated RNA was treated with RQ1 RNase-Free DNase following the manufacturer’s instructions (Promega, Fitchburg, WI, United States). RNA concentration was determined using a NanoDrop 2000 spectrophotometer (Thermo Fisher Scientific, Wilmington, DE, United States), and RNA integrity was checked in 1.0% agarose gel electrophoresis stained with ethidium bromide (1 μg mL^−1^). First-strand cDNA was synthesized from 1 μg of total RNA with the GoScript^TM^ Reverse Transcription System (Promega, Fitchburg, WI, United States), according to the manufacturer’s instruction. RT-qPCR was carried out in the StepOnePlus System (Applied Biosystems, Foster City, CA, United States) using GoTaq^®^qPCR Master Mix (Promega, Fitchburg, WI, United States).

Gene-specific primers of the cell wall and drought-stress responsive genes used in RT-qPCR analyses are listed in Supplementary Table 1. Analyses of transgene expression level (*n* = 3 ± standard error) were conducted using rice eukaryotic elongation factor-1α gene (Accession No. AK061464) as endogenous control to normalize the cDNA variance between samples (Martins et al., 2018).

### Transgene Copy Number Estimation in *ShDJ* Lines

To confirm the copy number of T-DNA inserted in the transgenic lines, genomic DNA from leaves was isolated using the cetyltrimethylammonium bromide (CTAB) method (Aljanabi et al., 1999). *ShDJ* copy number was evaluated in primary transformants (T_0_) by PCR using Taqman^®^Assay technology (Applied Biosystems, Foster City, CA, United States). The *hpt*II and sucrose phosphate synthase (*SPS*) primers and TaqMan probes were synthesized by Applied Biosystems (Foster City, CA, United States) and used in all analyses (Supplementary Table 1) (Ding et al., 2004). RT-qPCR and thermal profile reactions were performed as described by Martins et al. (2018) and the transgene copy number was determined as described by Mason et al. (2002) method.

### Transgenic Rice Plants Under Water Deficit Conditions

Transgenic rice seedlings were transferred to pots (5 L for T_1_; and 3 L for T_4_ plants) containing a mixture (1:1, v/v) of soil and substrate (Carolina Soil, Santa Cruz do Sul, Brazil) and grown under greenhouse conditions in Ribeirão Preto, Brazil (21°11′ S, 47°48′ W). Each pot contained one WT rice and one transgenic rice seedling, to ensure they were exposed to the same levels of water availability (Verslues et al., 2006). During the experimental period, the maximum photosynthetically active radiation (PPFD) and average air temperature were 1,567 μmol m^−2^ s^−1^ and 26.8 ± 8.8°C for T_1_ plants, and 1,691 μmol m^−2^ s^−1^ and 27.2 ± 3.1°C for T_4_ plants. Those environmental conditions were monitored with a Watch Dog 1450 Micro Station (Spectrum Technologies, Aurora, IL, United States).

To evaluate drought tolerance at the whole-plant level, two treatments were imposed 45 days after sowing (DAS): the control condition (*n* = 4 ± standard error), in which plants were maintained well-watered through daily irrigation; and the water deficit condition, which was induced by water withholding. According to Hsiao et al. (1984), severe stress for rice plants is considered when there is visible leaf wilting. In fact, this occurred in two previous pilot experiments and we adopted leaf wilting as an index of stress level. The recovery capacity of plants previously exposed to severe stress was evaluated 24 h after rehydration. Then, four plants from each *ShDJ* line and WT plants were used to evaluate total dry mass and tillering. Pot weight was evaluated daily to monitor the water availability (Supplementary Figure 1).

To evaluate plant survival under greenhouse conditions, 1-month old T_4_ progeny seedlings were maintained in substrate (Carolina Soil, Santa Cruz do Sul, Brazil) under well-watered. The water was withheld from for 5 days, which was sufficient to cause leaf wilting in transgenic rice overexpressing *ShDJ*. Then, plants were re-watered for two days, recovery was evaluated, and survival rates were estimated based on the percentage of survival in relation to all plants tested.

### Cell Wall Composition and Saccharification Analyses

Cell wall analyses were carried out in plants maintained under well-watered and water deficit conditions. Analyses were performed in shoots (stem and leaves) of T_4_ transgenic lines and WT plants, with four biological replicates each. Cell wall polysaccharides were evaluated as described by Chen et al. (2002) and the soluble and insoluble lignin contents (Klason’s Methods) were determined following the Tappi protocol UM-250 (TAPPI, 1985). The saccharification ratio was measured as described by methodology of Brown and Torget (1996), with modifications as described by Martins et al. (2018).

### Statistical Analyses

The experimental design was randomized in a 2 × 2 factorial scheme, with variation in terms of *ShDJ* (transgenic lines and WT plants) and water regimes (well-watered and water deficit). Data were subjected to analyses of variance (ANOVA) and when statistical significance was detected, the mean values were compared using a *t-test* (*P <* 0.05) with SAS statistical software (version 9.2; SAS Institute, Inc., Cary, NC, United States).

## Results

### *ShDJ* Gene Expression in Sugarcane Genotypes With Contrasting Drought Tolerance

The global expression analyses of ‘IACSP94-2094’ (drought-tolerant) and ‘IACSP97-7065’ (drought-sensitive) sugarcane (*Saccharum* spp.) genotypes were performed using microarray and RNA-seq assays in leaves provided from field and greenhouse experiments, respectively (Oliveira, 2012). Further details about the experiments and methodologies are described in Andrade et al. (2016).

**FIGURE 1:**
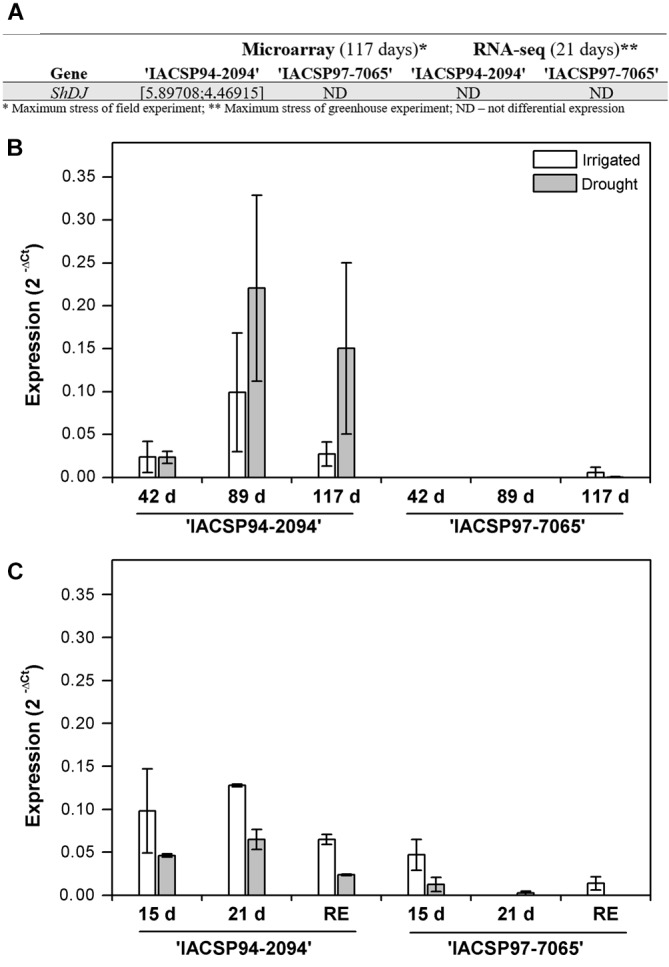
Quantitative polymerase chain reaction (PCR) analyses of the *ShDJ* gene in leaves of IACSP94-2094 and IACSP97-7065 sugarcane genotypes subjected to water deficit. **(A)** Screening for the *ShDJ* gene in global gene expression analyses using microarray and RNA-seq. **(B)** Field experiment, with evaluations 42, 89, and 117 days after last rainfall. **(C)** Greenhouse experiment, with evaluations after 15 and 21 days of water deficit and after 9 days of soil rehydration (recovery). The expression profile was evaluated by determined the difference in Ct between *ShDJ* and *UBQ1*, according to 2^−ΔCt^ (Livak and Schmittgen, 2001). In **(B,C)**, data represent the mean values [*n* = 3 ± standard error (SE)].

Among several differentially expressed candidate genes, the expression of dirigent-jacalin gene (SUCEST accession no. SCJLLR1103A10), here named *ShDJ* (*Saccharum* hybrid *Dirigent-Jacalin*) increased when the tolerant genotype was subjected to water deficit under field conditions (Figure 1A). In order to evaluate *ShDJ* expression, RT-qPCR assays were performed for each genotype, using the same leaf tissues (leaf +1, i.e., the first fully expanded leaf with visible ligule) used for the microarray and RNA-seq assays and sampled in both field (42, 89, and 117 days after the last rainfall) and greenhouse (15 and 21 days of water deficit and after 9 days of recovery) experiments.

Under field conditions, *ShDJ* transcripts were not detected 42 and 89 days after the last rainfall in ‘IACSP97-7065’ (Figure 1B). Conversely, *ShDJ* transcripts were detected at all time points in ‘IACSP94-2094,’ with drought-stressed plants presenting higher transcript levels than irrigated plants after 89 and 117 days of water deficit conditions (Figure 1B). Under greenhouse conditions, *ShDJ* expression responded to drought in the ‘IACSP94-2094’ genotype, with lower transcript abundance under drought conditions compared with irrigated conditions. In ‘IACSP97-7065,’ the *ShDJ* transcript was not detected after 21 days of water deficit and after rehydration (Figure 1C). Based on these results, the role of the *ShDJ* gene on drought tolerance was further investigated by sequence and phylogenetic analyses, followed by cloning and rice heterologous overexpression.

### Phylogenetic Analyses of *ShDJ*

**FIGURE 2:**
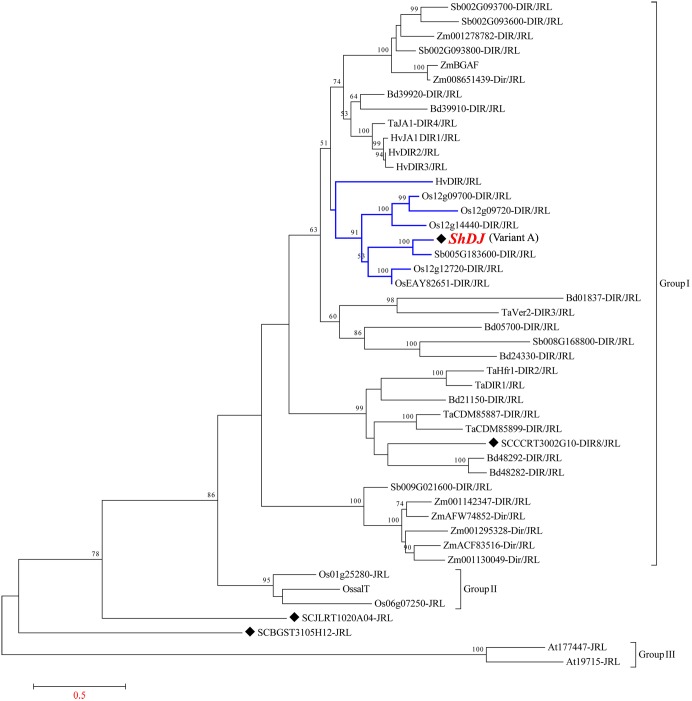
Phylogenetic relationship and multiple sequence alignment of the *ShDJ* protein with other *Dirigent-jacalin* (DJs). Phylogenetic analyses of the *ShDJ* protein and DJs protein sequences from sorghum (Sb), rice (Os), maize (Zm), *Arabidopsis thaliana* (At), *Setaria italica* (Si), *Panicum virgatum* (Pavir), and *Hordeum vulgare* (Hv) were generated using the neighbor-joining (NJ) method in MEGA6. Bootstrap values greater than 50% (1,000 replicates) are shown for nodes in the tree. Black symbols indicate the sugarcane DJs.

A total of 46 annotated amino acid sequences homologous to *ShDJ* were obtained from various plant species (Supplementary Table 2) and used to construct a phylogenetic tree (Figure 2), aiming to investigate the evolutionary history of DJ proteins. Two SAS from the SUCEST database were identified as a chimeric DJ protein, and the sequences were named DIR4/JRL (SCJLLR1103A10, corresponding to *ShDJ*) and DIR8/JRL (SCCCRT3002G10) according to Nobile et al. (2017). The phylogenetic tree presented three major groups composed of DJ and *Jacalin* sequences (Figure 2). The DJ proteins comprised the major group I exclusive for monocot plants, while group II and group III were formed by proteins with a jacalin domain from rice and *Arabidopsis*, respectively (Figure 2). Two JRL sugarcane sequences (SCJLRT1020A04-JRL and SCBGST31051112-JRL) were positioned between group II and group III (Figure 2).

The sequence similarity search revealed several proteins with high identity to *ShDJ*. The phylogenetic tree showed that the *ShDJ* protein was close to rice (Os12g12720-DIR/JRL, OsEAY82651-DIR/JRL, Os12g14440-DIR/JRL, Os12g09720-Dir/JRL, Os12g09700-DIR/JRL), *Hordeum vulgare* (HvDIR/JRL) and sorghum (Sb005G183600-DIR/JRL) (Monocot JRL, Figure 2). *ShDJ* was closest to sorghum (Sb005G183600), showing high confidence level (100% bootstrap). According to blast2, *ShDJ*, and Sb005G183600 shared 85% identity.

### Isolation and Characterization of *ShDJ*

The *ShDJ* gene sequence in the SUCEST database is incomplete; then the full-length sequence was revealed by using SMARTer^TM^ RACE cDNA Amplification Kit (Clontech, Mountain View, CA, United States). The CDS of the *ShDJ* gene was successfully amplified from the 5′ and 3′ RACE libraries of ‘IACSP94-2094’ leaves. The cloned *ShDJ* full-length sequences revealed two probable allelic variants A and B (Supplementary Figure 2). Variant B (MK000561) presented four additional nucleotides compared with to variant A (MK000560), thereby changing the ORF, to encode a truncated protein (Supplementary Figure 2). Therefore, variant A was used for vector construction and for functional genomic analyses. The isolated sequence exhibited a 924 bp ORF encoding a polypeptide of 308 amino acids with a predicted molecular mass of 76.09 kD and isoelectric point (pI) of 5.07 calculated with the ExPASy compute pI/Mw tool^4^. The 308 amino acids encode a dirigent (amino acids 29–148) and a jacalin (amino acids 175–306) domain according to a BLAST protein–protein search of Pfam^5^.

### Overexpression of the *ShDJ* Gene Increases the Drought Tolerance of Rice Plants

**FIGURE 3:**
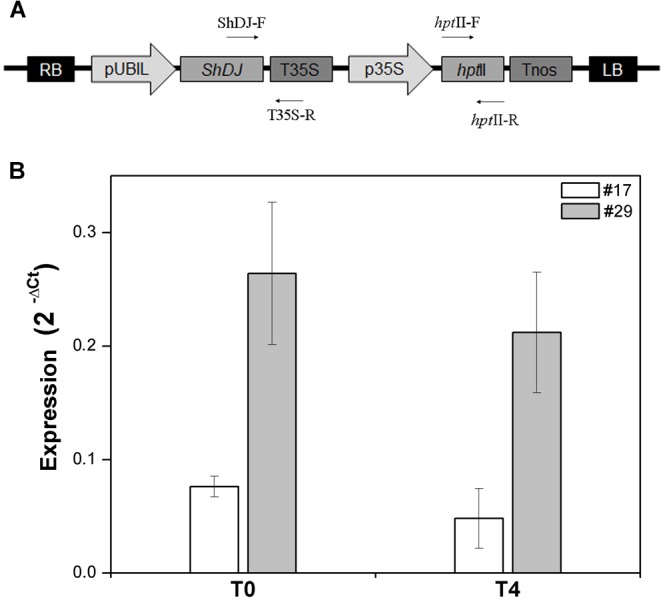
Molecular characterization of *ShDJ* lines. **(A)** Schematic representation of the T-DNA region of the binary vector *pUBIL::ShDJ.* RB, right border; LB, left border; pUBIL, maize ubiquitin promoter; T*nos*, nopaline synthase terminator; p35S, 35S promoter; *hptII*, hygromycin phosphotransferase gene (selectable marker); T35S, 35S terminator. **(B)** Analyses of *ShDJ* expression in rice plants of T_0_ and T_4_ generations. Abundance of the *ShDJ* transcripts in lines #17 and #29 was evaluated by real time quantitative polymerase chain reaction (RT-qPCR) analyses (*n* = 3 ± standard error) using gene-specific primers for *ShDJ*. Analyses used the 2^−ΔCt^ method (Livak and Schmittgen, 2001), in which ΔCt represents the relative quantification of a target gene and a reference gene (elF-1α).

To investigate the role of *ShDJ* in drought tolerance, a *ShDJ*-overexpression vector was constructed under control of the maize ubiquitin promoter and used for rice transformation (*Oryza sativa* L.) (Figure 3A). Thirty independent lines were produced, hereafter called rice *ShDJ* lines, and confirmed as positive transformants by conventional PCR in T_0_ plants (data not shown). The *ShDJ* lines grew to maturity for setting seeds, and no morphological alterations were observed under normal growth conditions. Variation in the transgene expression of *ShDJ* lines was evaluated by RT-qPCR, ranging from 0.003 to 0.617 (Supplementary Figure 3). Among *ShDJ* lines, line #24 showed the highest *ShDJ* expression and line #5 showed the lowest expression, whereas no *ShDJ* expression was detected in WT plants (Supplementary Figure 3), as expected.

Analyses of transgene expression were followed by evaluation of copy number as described by Mason et al. (2002) using TaqMan methodology. Twelve *ShDJ* lines showing differential expression of the *ShDJ* transgene (high, medium, and lower) were chosen, and the transgene copy number integrated into the genome ranged from 1 to 4 (Supplementary Figure 3). According to those results, T_1_ progeny from five independent transgenic lines (#1, #8, #17, #24, and #29) exhibiting different expression levels, but carrying one transgene copy were selected to evaluate the role of *ShDJ* under water deficit conditions. In a preliminary experiment, five *ShDJ* lines of T_1_ progeny were evaluated for drought tolerance under greenhouse conditions. Following this initial screening, transgenic lines #17 and #29 were further investigated considering T_4_ progeny (Figure 3B). Lines #17 and #29 were chosen due to their drought tolerance and biomass accumulation, and represent the greatest contrast to WT plants.

**FIGURE 4:**
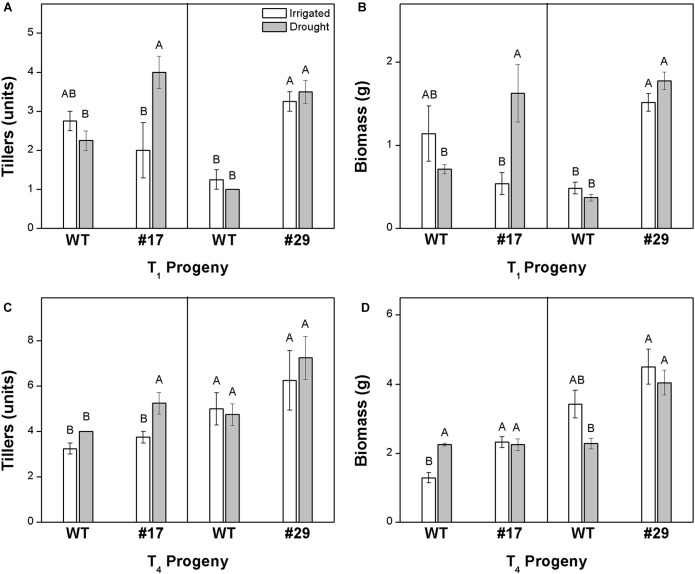
Tillering **(A,C)** and biomass **(B,D)** of *ShDJ* transgenic lines #17 and #29 in T_1_ **(A,B)** and T_4_ **(C,D)** progenies. Mean values (*n* = 4) ± SE. Different letters indicate significant differences between plants and water conditions (*t*-test, *P <* 0.05).

Leaf wilting was observed after 12 and 8 days of water deficit in T_1_ and T_4_ progenies, respectively. When evaluating T_1_ progeny, line #17 exhibited higher biomass and tillering compared with the WT plants under water deficit conditions, while line #29 exhibited higher vigor than WT plants under both water regimes (Figure 4A,B). In T_4_ progeny, line #29 showed higher biomass and line #17 presented higher tillering compared with WT plants under water deficit conditions (Figure 4C,D). In general, tillering was improved in *ShDJ* transgenic line #17 under water deficit conditions, regardless of progeny. Among the T_1_ progeny, line #29 showed a large increase in biomass under both well-watered and water deficit conditions (Figure 4B). No differences were found in the seed size of transgenic lines under varying levels of water availability (Supplementary Figure 4).

**FIGURE 5:**
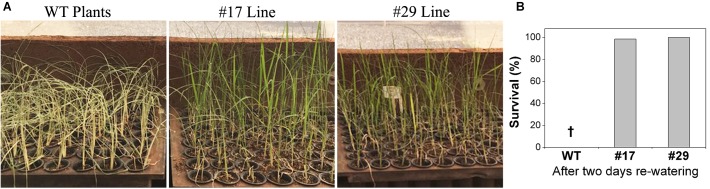
Overexpression of *ShDJ* increased the survival of T_4_ transgenic rice seedlings under drought stress. **(A)** Seedlings of WT and lines #17 and #29 2 days after re-watering. **(B)** Survival rate of WT and transgenic rice seedlings after drought. Survival rates were calculated as the ratio of surviving plants to the total number of plants after re-watering.

To investigate the role of *ShDJ* in plant survival under drought, water was withheld from rice seedlings for 5 days. The survival rate (%) was determined 2 days after re-watering. The survival rate of the transgenic lines was higher than that of the WT plants (Figure 5). Notably, none of the WT plants survived under water deficit conditions (0%), while only one transgenic plant (out of 68), line #17 failed (98,5%), and all plants of line #29 survived (100%) following rehydration. These results clearly revealed the role of the *ShDJ* gene on the drought tolerance of transgenic plants.

In addition, *ShDJ*-overexpression was evaluated for salinity tolerance. Although a recent report showed that drought and salinity tolerance share the same complex regulatory processes involved in cellular homeostasis (for review, see Golldack et al., 2014), no differences were observed in *ShDJ* transgenic lines when compared with WT plants under salt stress (Supplementary Figure 5). All the experiments have been adhered the standard biosecurity and institutional safety procedures, following requirements and biosafety procedures of National Technical Commission on Biosafety (CTNBio) to GMOs manipulation biosafety level 1.

### *ShDJ*-Overexpression in Rice Causes Changes in the Cell Wall Composition

**FIGURE 6:**
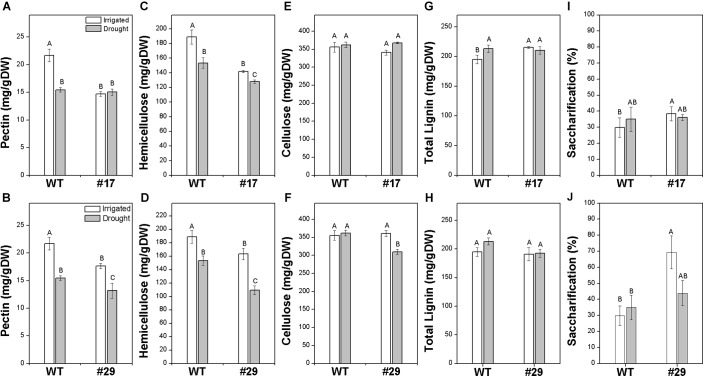
Effects of *ShDJ* overexpression on the cell wall composition and saccharification efficiency of transgenic rice lines #17 **(A,C,E,G,I)** and #29 **(B,D,F,H,J)** under varying levels of water availability. **(A,B)** Pectin; **(C,D)** hemicellulose; **(E,F)** cellulose; **(G,H)** total lignin; **(I,J)** saccharification. Evaluations considered the entire plant shoot of T_4_ progeny. Each histogram is the mean value (*n* = 4) ± SE. Different letters indicate significant differences between plants and water conditions (*t*-test, *P <* 0.05).

Besides increasing drought tolerance, the overexpression of *ShDJ* was investigated considering possible modifications in cell wall composition, such as lignin and polysaccharides. Cell wall composition was evaluated in T4 progeny and WT plants, considering the entire plant shoots. *ShDJ* and WT plants showed different ranges of pectin, hemicelluloses, cellulose, and total lignin contents. Under well-watered conditions, lines #17 and #29 presented a significant decrease in pectin and hemicellulose compared with WT plants, with reductions ranging from ∼18–30 to ∼13–25%, respectively (Figure 6A–D). Under water deficit conditions, no significant difference in pectin was observed in line #17, whereas a decrease in hemicellulose content was observed in both transgenic lines compared with WT (Figure 6A–D). *ShDJ* overexpression did not affect cellulose content in line #17 under both water regimes, whereas the cellulose content of line #29 was lower (−14%) than that in WT plants under water deficit conditions (Figure 6E,F). Regarding lignin, there was a significant increase in line #17 compared with WT plants (Figure 6G). Conversely, *ShDJ* overexpression in line #29 did not affect total lignin under both water regimes (Figure 6H). We also found significant increases in saccharification efficiency in lines #17 and #29, ranging from 28% (line #17) to 132% (line #29) compared with WT plants under well-watered conditions (Figure 6I,J).

### Expression Analyses of Secondary Cell Wall and Drought-Stress Responsive Genes in *ShDJ* Transgenic Lines

To investigate the contribution of constitutive *ShDJ* expression in transgenic rice lines, the expression pattern of genes involved in the regulation of the secondary cell wall (*OsMYB58/63* and *OsNST1/2*) and water deficit response (*OsP5CS, OsLea3, OsGRAS23*, and *OsbZIP23*) was assessed. Gene expression pattern of leaves and stems of T_4_ plants were compared under well-watered conditions.

**FIGURE 7:**
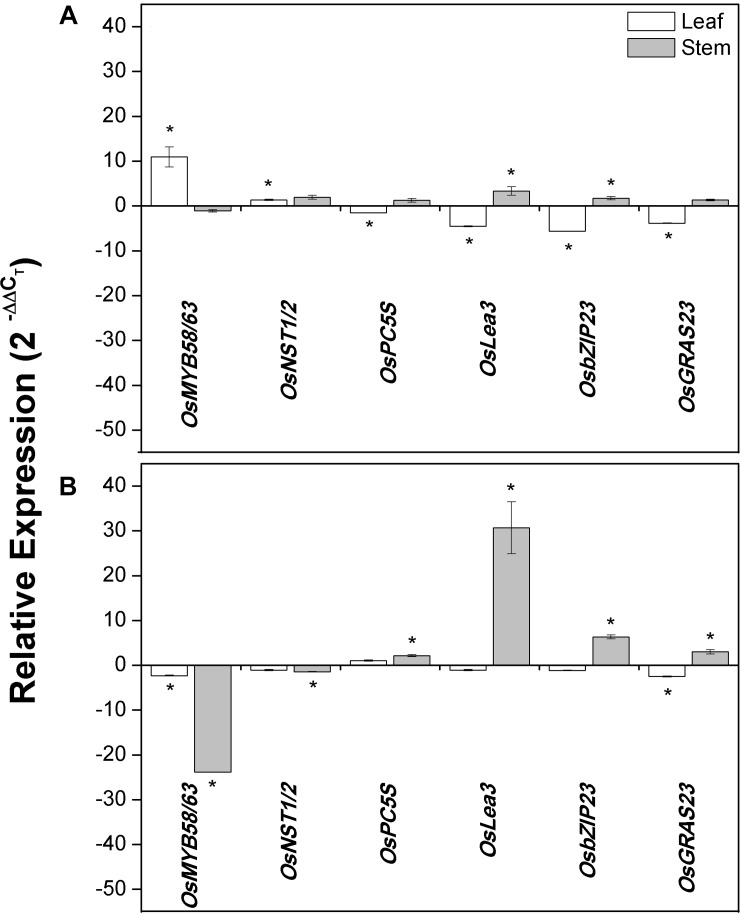
*ShDJ* modified the expression of cell wall and drought-related genes in leaves and stems of transgenic rice lines **(A)** #17 and **(B)** #29. Expression profiles of *OsMYB58/63*, *OsNST1/2*, *OsP5CS*, *OsLea3*, *OsbZIP23*, and *OsGRAS23* genes observed by RT-qPCR analyses in T_4_ plants were maintained under irrigated conditions. Expression in transgenic lines is relative to that in WT plants using the 2^−ΔΔCt^ method (Livak and Schmittgen, 2001). Data are mean values (*n* = 3) ± SE, and asterisks on the top of each bar indicate significant differential expression between lines and WT as determined using REST© software (5% significance).

The expression of *OsMYB58/63* (Ambavaram et al., 2011; Noda et al., 2015) and *OsNST1/2* (Ambavaram et al., 2011) were significantly increased in the leaves of line #17 (11- and 1.3-fold increases, respectively) compared with WT plants (Figure 7A). In line #29, *OsMYB58/63* expression was down-regulated in leaves (2.3-fold) and stems (24-fold), while *OsNST1/2* expression was reduced only in stems (1.5-fold) (Figure 7B).

There was a significant decrease in *OsP5CS* (Zhang and Chen, 2017) expression in leaves of line #17 compared with WT plants (1.5-fold), while expression was up-regulated (2.2-fold) in stems of line #29 (Figure 7). *OsLea3* (Zhang and Chen, 2017) expression was up-regulated in stems, with lines #17 and #29 showing 3.4- and 31-fold higher expression compared with WT plants (Figure 7). Decreased expression of *OsLea3* was observed in leaves of line #17 (Figure 7A). Transcription factors related to drought stress via ABA-independent (*OsGRAS23*; Xu et al., 2015) and ABA-dependent (*OsbZIP23*; Xiang et al., 2008) pathways were also evaluated and found to present a similar expression profile. While the transcript abundance of *OsGRAS23* was clearly decreased in leaves of lines #17 (3.9-fold repression) and #29 (2.5-fold repression) compared with WT, it was increased (3-fold) in stems of line #29 (Figure 7). *OsbZIP23* expression was up-regulated in stems of lines #17 (1.7-fold) and #29 (6.4-fold) and down-regulated (5.61-fold) in leaves of line #17 (Figure 7).

## Discussion

In the present study, expression of the *ShDJ* gene was systematically investigated in sugarcane genotypes with contrasting levels of drought tolerance and characterized in drought stress through the heterologous overexpression of *ShDJ* in rice. Rice (*Oryza sativa*) is widely used as a model plant for functional analyses of monocots. Therefore, we used rice for functional analyses of sugarcane genes as it is a monocot plant evolutionarily close to sugarcane and has well-established transformation protocols. Furthermore, rice is a diploid species with a small genome (about 7–8 fold smaller than sugarcane genome), and with a short life cycle (up to three generations per year). Our recent results of sugarcane genes overexpressed/silenced in rice have shown linearity with those obtained from the overexpression/silencing in sugarcane (proof of concept – data not published). Indeed, rice is a good model for functional analyses of sugarcane genes evolved in drought stress, although we need to validate *ShDJ* in sugarcane.

Although genes encoding DJ proteins are widely present in monocot plants (Schutter and Van Damme, 2015), their biological functions are still poorly understood. The sugarcane genome contains at least four DJ groups comprising seven non-redundant sequences (Nobile et al., 2017), and *ShDJ* responded strongly to drought stress in ‘IACSP94-2094,’ a drought-tolerant genotype (Figure 1). Herein, RT-qPCR results validated transcriptome data for the *ShDJ* gene during drought stress under both field and greenhouse conditions.

Previous studies with proteins containing the dirigent-jacalin domain have demonstrated the involvement of this domain in pathogen resistance (Williams et al., 2002; Subramanyam et al., 2006; Ma et al., 2010; Song et al., 2014). Based on a search of EST and microarray databases, only two studies in wheat have reported the responsiveness of DJ genes to drought stress (Song et al., 2014; Kumar et al., 2018). Song et al. (2014) showed that the expression of DJ genes was induced in response to PEG treatment, including *TaJRL6* (identified in the phylogenetic tree as TaDIR1/JRL) present in group I (Figure 2). Based on their results, those authors suggested that JRL proteins seem to play an important role in plant adaptation under stressful conditions. In addition, Kumar et al. (2018) identified DJ genes, including Ta.188.1.S1_at (TaDIR1/JRL), using a microarray database, which were strongly induced under drought in drought-tolerant wheat genotype. Although JRL wheat proteins in these studies fall within group I of the DJ proteins, the probably orthologous groups are not evident among JRL genes. According to Walley and Dehesh (2010) and Xiao et al. (2013), biotic and abiotic stress pathways in plants are regulated by cross-talk between signaling networks. Therefore, DJ genes are inducible by both biotic and abiotic stresses, suggesting multiple roles of DJ genes in plants.

### *ShDJ*-Overexpression in Rice Promotes Drought Tolerance, Biomass Accumulation, and Improves Saccharification

In this study, the role of the *ShDJ* gene in the water deficit response was characterized for the first time. Transgenic rice lines overexpressing the *ShDJ* gene were generated and phenotyped in response to water deficit stress under greenhouse conditions. Large differences in total biomass production (tiller number and total dry mass) were found between *ShDJ* lines and WT plants in both generations evaluated (Figure 4). Under drought, *ShDJ* lines maintained growth and development, resulting in higher biomass in lines #17 and #29, while a significant reduction in total dry mass occurred in WT plants (Figure 4). In contrast, the survival ratio test, revealed that the *ShDJ* lines showed strong tolerance to water deficit stress (Figure 5). These results strongly suggest that *ShDJ* plays a role in drought tolerance as well as in the growth and development of transgenic plants. The role in the growth and development of rice plants were suggested by Jiang et al. (2007). The authors evaluated the OsJAC1 gene promoter fused to the GUS reporter gene (pOsJAC1::GUS) and presents a constitutive gene expression in rice. However, when the OsJAC1 gene was driven by the maize constitutive promoter (Ubi::OsJAC1), the plants showed a reduction in coleoptile and stem elongation (Jiang et al., 2007). Similarly to our study, Ambavaram et al. (2014) observed that overexpression of the transcription factor HYR in rice resulted in an increase in grain yield and biomass accumulation, regardless of water availability. Likewise, Karaba et al. (2007) showed that constitutive expression of the HARDY *Arabidopsis* gene in rice, improved drought tolerance and increased biomass production. Recently, Bi et al. (2018) demonstrated that the overexpression of *Arabidopsis* SHN1 in wheat increased biomass production under drought stress conditions compared with WT plants. Therefore, a series of complex traits relevant to biomass or yield, such as survival rate, tillering, and dry mass have been used to evaluate the drought tolerance (Fang and Xiong, 2015). In fact, these traits are important criteria for phenotyping drought stress tolerance in crop breeding (Mitra, 2001).

To investigate how *ShDJ* would affect cell wall composition and saccharification efficiency, rice shoots of T4 progeny were examined. Biochemical analyses were also performed to elucidate the contribution of *ShDJ*-overexpression to biomass saccharification, since the recalcitrance of cell walls to hydrolysis represents the major bottleneck for the E2G industry (Himmel et al., 2007). Under well-watered conditions, there was a significant reduction in pectin and hemicellulose in both transgenic lines compared with WT plants, whereas the cellulose content was unchanged under well-watered conditions (Figure 6). Under drought, there was a decrease in hemicellulose content in line #17 and we observed a significant reduction in pectin, hemicellulose, and cellulose content in line #29 (Figure 6). The same response to drought has been reported in wheat coleoptile (Wakabayashi et al., 1997), squash hypocotyls (Sakurai et al., 1987a,b), maize leaves (Acevedo et al., 1971), grape leaves (Sweet et al., 1990), corn stover, mixed grasses, and Miscanthus (Emerson et al., 2014). In those studies, drought had a negative impact on growth, and therefore on total biomass yield. Similarly, Lionetti et al. (2010) showed reduced polysaccharide content due to genetic modification of the cell wall inhibited plant growth. However, in our study, modulation of cell wall did not adversely affect the growth of transgenic lines, as they displayed normal plant phenotypes and even increases in biomass production (Figure 6).

Pectin and hemicellulose link cellulose and lignin, and this interaction has a major role in cell wall recalcitrance (Himmel and Bayer, 2009). Changes in cell wall polysaccharides may cause cell wall loosening (Thompson, 2005), and this loosening phenomenon, as proposed by Moore et al. (2008), is related to increases in cell wall elasticity (Gall et al., 2015), promoting polysaccharides accessibility and increasing biomass solubilization (Phitsuwan et al., 2013). Based on our results, the reduction of pectin and hemicellulose content in *ShDJ* lines compared with WT plants suggests less interaction between cell wall polysaccharides and a significant improvement in saccharification efficiency (Figure 6). Herein, we provide strong evidence that modifications in cell wall composition affect biomass recalcitrance, thus increasing saccharification. This may due to efficient enzymatic action, reducing interactions among pectin, hemicellulose, and lignocellulose components (Himmel et al., 2007; Scheller and Ulvskov, 2010). Removal of hemicellulose (Qing et al., 2010; Shin et al., 2010) and pectin (Lionetti et al., 2010; Chen and Peng, 2013; Biswal et al., 2014) from cell wall enhances saccharification. Downregulation of the GAUT12.1 gene (hemicellulose biosynthesis) in *Populus deltoides* by RNAi led to a reduction in recalcitrance due to decreases in hemicellulose and pectin contents, while lignin content was unchanged with a significant increase in plant growth (Biswal et al., 2015). Our results suggest that *ShDJ* acts by altering pectin and hemicellulose metabolism and support our hypothesis that overexpression of *ShDJ* increases drought tolerance and causes cell wall modifications, with benefits for plant growth saccharification in transgenic lines.

### Expression Profiles of Genes Related to Cell Wall Composition and Water Deficit Response

Several studies have shown that changes in gene expression and regulatory genes (transcription factors) are involved in the activation of drought response and tolerance (Bartels and Sunkar, 2005). As *ShDJ*-overexpression could have affected many genes, we examined the molecular mechanisms modified in transgenic lines under well-watered conditions, considering genes involved in cell wall biosynthesis and water deficit tolerance.

Transcription factors *OsMYB58/63* (Ambavaram et al., 2011; Noda et al., 2015) and *OsNST1/2* (Ambavaram et al., 2011) regulate transcription of secondary cell wall genes. The present study evaluated *OsMYB58/63* and *OsNST1/2* genes to confirm that *ShDJ*-overexpression affected the expression of cell wall genes in transgenic lines. In addition, Zhou et al. (2009) described *MYB58* and *MYB63* as transcriptional activators of the lignin biosynthetic pathway during secondary cell wall formation in *Arabidopsis*. However, we observed a contrasting expression profile of both genes in the transgenic lines evaluated. Expression of cell wall genes was induced in leaves of line #17, which was consistent with the higher lignin content in this line compared with WT plants (Figure 7). Conversely, the reduced expression of genes did not alter lignin content in line #29 (Figure 7). A reduction in expression was observed by Ambavaram et al. (2011), and *AtSHN*-overexpression in rice directly repressed *OsMYB58/63* and *OsNST1/2* in leaves and stems, resulting in a large reduction in lignin content. Recently, Martins et al. (2018) demonstrated that the overexpression of sugarcane ShSHN1 in rice repressed *OsMYB58/63* and *OsNST1/2* in leaves and tillers followed by a decrease in lignin content in the transgenic lines and improvement of saccharification efficiency. Interestingly, ShSHN1 rice transgenic lines showed an increase in biomass production when compared with WT plants. Altogether, those results support the conclusion that *ShDJ* modulates the expression of *OsMYB58/63* and *OsNST1/2* genes related to cell wall formation. However, the relation between expression and cell wall components needs to be further studied and understood to uncover the underlying processes leading to biomass accumulation and how *ShDJ* affects them.

To date, various signaling pathways have been reported to be involved in drought tolerance in rice, including responsive genes and transcription factors. Earlier reports showed that activation of these genes improved drought tolerance (Xiao et al., 2007; Xiang et al., 2008; Xu et al., 2015; Lou et al., 2017; Zhang and Chen, 2017). LEA and P5CS proteins have crucial roles in osmotic adjustment in many plants, protecting them from damage caused by environmental stresses, such as drought (for review, see Fang and Xiong, 2015). Conversely, transcription factors play important roles in the transcriptional regulation of stress-related genes (Shinozaki et al., 2003).

In the present report, we found that *ShDJ*-overexpression upregulated the expression of drought-related genes and transcription factors in rice stems. *OsP5CS* and *OsLea3* showed a similar expression profile, with increased expression levels observed in stems and decreased expressed in leaves in both transgenic lines compared with WT plants (Figure 7). According to Zhang and Chen (2017), the overexpression of *OsNRRB* activated the expression of *OsLea3* and *OsP5CS*, while the expression of *OsbZIP23* was repressed in leaves of well-watered plants, as reported herein (Figure 7). Xu et al. (2015) observed that expression of the transcription factor *OsGRAS23* was induced by drought stress, and showed that *OsGRAS23*-overexpression is involved in abiotic stress responses, plant growth, development, and phytohormone signal transduction (e.g., JA) in rice. In addition, transgenic rice overexpressing *OsLEA3* (Xiao et al., 2007) and *OsbZIP23* (Xiang et al., 2008) presented a significant improvement in drought tolerance. In contrast, *OsSAPK2*-silenced plants, there were no differences in the expression levels of *OsLea3*, *OsP5CS*, and *OsbZIP23* in leaves as compared with WT plants under well-watered conditions (Lou et al., 2017). Additionally, the overexpression of *ShDJ* increased the expression of *OsbZIP23* and *OsGRAS23* transcription factors in stems, revealing changes in ABA-dependent and ABA-independent signaling pathways, respectively. This suggests that drought tolerance can be genetically regulated by both hormonal pathways. Therefore, our results indicated that overexpression of the *ShDJ* gene in rice may impact the steady-state of transcription of stress–response genes, which may improve drought tolerance and promote changes in secondary cell walls.

## Conclusion

In this study, we revealed that overexpression of *ShDJ* gene contributed to drought tolerance, maintaining plant growth and development of transgenic lines under conditions of low water availability. The *ShDJ* gene proved to be a good candidate for genetic transformation of plants to improve drought tolerance, using only one gene as a target. While further experimentation is needed under field conditions, our results highlight an interesting pathway for enhancing productivity within a sustainable context, where water is a limiting factor. Given that *ShDJ*-overexpression promotes improvement in saccharification efficiency in rice, our findings are of special interest for bioenergy production using sugarcane. The development of new sugarcane cultivars focused on first (E1G) and second-generation (E2G) ethanol, and on the co-generation of energy, should promote environmental and economic benefits, increasing crop yield per area planted.

## Author Contributions

JFCON, AF, RVR, and SC performed field and greenhouse sugarcane experiments. LMA, JFCON, PMN, RFP-J, CF, and MSB conducted global gene expression data analyses and RT-qPCR assays. LMA, TRB, JS, and MHSG performed the cloning and vector construction for plant transformation. LMA, PERM, SDC, and RVR performed the drought assays with transgenic lines. JPPL and PM conducted the cell wall assays. LMA and DP performed statistical analyses. LMA and SC wrote the manuscript. PMN, MSB, and RVR provided intellectual input and revised the manuscript. All authors read and approved the final manuscript.

## Conflict of Interest Statement

The authors declare that the research was conducted in the absence of any commercial or financial relationships that could be construed as a potential conflict of interest.

## Footnotes

1 http://sucest-fun.org/

2 http://www.ncbi.nlm.nih.gov/

3 https://phytozome.jgi.doe.gov/

4 https://web.expasy.org/compute_pi/

5 https://pfam.xfam.org/
