# Supplementary material for: Biomass Accumulation and Cell Wall Structure of Rice Plants Overexpressing a Dirigent-Jacalin of Sugarcane (ShDJ) Under Varying Conditions of Water Availability
Source: Front Plant Sci. 2019 Feb 13;10:65. doi: 10.3389/fpls.2019.00065 (PMC6381051; doi:10.3389/fpls.2019.00065)
Supplement: Supplementary file 1 [file Data_Sheet_1.docx]

Supplementary Material

Biomass accumulation and cell wall structure of rice plants overexpressing a dirigent-jacalin of sugarcane (*ShDJ*) under varying water availability

Larissa Mara Andrade, Rafael Fávero Peixoto-Junior, Rafael Vasconcelos Ribeiro, Paula Macedo Nóbile, Michael Santos Brito, Paulo Eduardo Ribeiro Marchiori, Samira Domingues Carlin, Alexandre Palma Boer Martins, Maria Helena S Goldman, Juan Pablo Portilla Llerena, Caroline Fregonesi, Dilermando Perecin, João Felipe Carlos de Oliveira Nebó, Antonio Figueira, Thiago Romanos Bennatti, Jorge da Silva, Paulo Mazzafera, Silvana Creste^*^

*** Correspondence:** Silvana Creste: screste@iac.sp.gov.br

# Supplementary Table

| **Supplementary Table 1.** List of primer sequences and respective gene names, accession numbers, and amplicon size (bp). | | | |
| --- | --- | --- | --- |
| **Gene** | **Accession** | **Sequence (5´- 3´)** | **Amplicon (bp)** |
| ***ShDJ* gene expression in sugarcane plants** | | | |
| *ShDJ* | SCJLLR1103A10 | F: CAGAGGGTTGTCACCATTCA  R: CATCTTGACACCATCGGACA | 152 |
| **Full-length amplification of *ShDJ* gene by RACE methodology** | | | |
| *ShDJ* | SCJLLR1103A10 | F: CAGAGGGTTGTCACCATTCA  R: CATCTTGACACCATCGGACA | |
| *ShDJ*_NESTED | SCJLLR1103A10 | F: GACATCACAGAGCCACCAAA  R: GCATCCAGGAACGTCCCGCT | |
| UPM | RACE | Long: CTAATACGACTCACTATAGGGCAAGCAG  TGGTATCAACGCAGAGT  Short: CTAATACGACTCACTATAGGGC | |
| UPM_NESTED | RACE | AAGCAGTGGTATCAACGCAGAGT | |
| **Rice transformation vector** | | | |
| attB1 | GCAGGCTTCACC-(~21 pb gene) | | |
| attB2 | AAGCTGGGTC-(~21 pb gene) | | |
| BP1 | GGGGACAAGTTTGTACAAAAAAGCAGGCTTC | | |
| BP2 | GGGGACCACTTTGTACAAGAAAGCTGGGTC | | |
| **Confirmation of transgenic rice** | | | |
| *hpt*II | *Hygromicin* | F: CACTGGCAAACTGTGATGGA  R: AGCGAACTGTGGACGAGAACT | 670 |
| *ShDJ*-F | *Dirigent-Jacalin* | F: AGAACTGGGGAGGGGAACA | 628 |
| T35S-R | 35S Terminator | R: ACTGGTGATTTTTGCGGACT |  |
| **Reference gene used for normalization evaluation in rice expression** | | | |
| elF-1α | AK061464 | F: AAGAACGGTGATGCTGGTATG  R: AACGACCAAGAGGAGGGTACT | 88 |
| **Expression of transgene in rice** | | | |
| *ShDJ* | SCJLLR1103A10 | F: AACTCCACCCAGTCGCAAAT  R: AGGAACCAGAGAGCCCATCAC | 91 |
| **Transgene copy number in rice** | | | |
| *SPS* | F: TTGCGCCTGAACGGATAT  R: CGGTTGATCTTTTCGGGATG | | |
| Probe *SPS* (fluorescent reporter FAM) | GACGCACGGACGGCTCGGA | | |
| *hpt*II | F: CGCAGCGATAGCATCCATAG  R: AGACCTGCCTGAAACCGAACT | | |
| Probe *hpt*II  (fluorescent reporter VIC) | CCGGCTGAAGAAC | | |
| **Genes related to *ShDJ* overexpression** | | | |
| *OsMYB5863* | Os04g50770 | F: GACCGAATCGTCCAGTGATG  R: CCGTCTTCGTATTGGATTGGT | 100 |
| *OsNST1/2* | Os08g02300 | F: ACAGAGACAAGACAAGTAAGAA  R: ATGGTGGATCTCTCTCTCTCTC | 83 |
| *OsP5CS* | Os05g38150 | F: AGTGGTGAATGGTGACAAGGA  R: ACCCCCAAAATCAATAATCCA | 192 |
| *OsLea3* | Os05g46480 | F: CCGTGAATGATTTCCCTTTG  R: ACGACCACCACTTCATACAGC | 120 |
| *OsGRAS23* | Os04g50060 | F: GCGTATCATCTTTGCTCTTTCAG  R: CCACAGGTCACACACGCTTAGA | 240 |
| *OsbZIP23* | Os02g52780 | F: TAGTCCGTGGGCTTGTCTCT  R: AATACAATACAGGTAGCAGGCAC | 132 |

ACT - Actin; UBQ – Ubiquitin; eF - Eukaryotic elongation factor; UBC - Ubiquitin-conjugating enzyme; *hpt*II – Hygromicin; SPS - Sucrose phosphate synthase.

**Supplementary Table 2.** Sequences used in the phylogenetic analyses of *Dirigent-Jacalin.*

| Gene | Accession/Locus | Protein (aa) | Database bank/ Literature |
| --- | --- | --- | --- |
| Sugarcane | | | |
| SCJLLR1103A10-DIR/JRL | SCJLLR1103A10 | 307 | SUCEST |
| SCCCRT3002G10-DIR/JRL | SCCCRT3002G10 | 307 | SUCEST |
| SCJLRT1020A04-JRL | SCJLRT1020A04 | 153 | SUCEST |
| SCBGST3105H12-JRL | SCBGST3105H12 | 147 | SUCEST |
| *Sorghum bicolor* | | | |
| Sb005G183600-DIR/JRL | Sobic.005G183600 | 303 | Phytozome |
| Sb002G093800-DIR/JRL | Sobic.002G093800 | 302 | Phytozome |
| Sb002G093700-DIR/JRL | Sobic.002G093700 | 305 | Phytozome |
| Sb002G093600-DIR/JRL | Sobic.002G093600 | 312 | Phytozome |
| Sb009G021600-DIR/JRL | Sobic.009G021600 | 304 | Phytozome |
| Sb008G168800-DIR/JRL | Sobic.008G168800 | 304 | Phytozome |
| *Oryza sativa* | | | |
| Os01g25280-JRL | Os01g25280 | 144 | Phytozome |
| Os06g07250-JRL | Os06g07250 | 183 | Phytozome |
| OsEAY82651-DIR/JRL | EAY82651.1 | 304 | NCBI |
| Os12g09700-DIR/JRL | Os12g09700 | 307 | Phytozome |
| Os12g14440-DIR/JRL | Os12g14440 | 306 | Phytozome/ Jiang et al. (2006) |
| Os12g12720-DIR/JRL | Os12g12720 | 260 | Phytozome |
| Dir/JRL-Os12g09720 | Os12g09720 | 258 | Phytozome |
| *salT* (JRL) | A2WPN7 | 145 | NCBI |
| *Zea Mays* | | | |
| Zm001278782-DIR/JRL | GRMZM2G402417 | 315 | Phytozome |
| ZmBGAF-DIR1/JRL | AAF71261 | 306 | NCBI/ Kittur et al. (2010) |
| ZmACF83516-Dir/JRL | GRMZM2G112238 | 322 | Phytozome |
| Zm001295328-Dir/JRL | GRMZM2G046520 | 325 | Phytozome |
| Zm008651439-Dir/JRL | GRMZM2G172204 | 306 | Phytozome |
| Zm001142347-DIR/JRL | GRMZM2G050412 | 325 | Phytozome |
| Zm001130049-Dir/JRL | GRMZM2G025959 | 324 | Phytozome |
| ZmAFW74852-Dir/JRL | GRMZM2G163406 | 312 | Phytozome |
| *Triticum aestivum* | | | |
| TaHfr1-DIR2/JRL | AAM46813 | 345 | NCBI/ Subramanyam et al. (2008); Song et al. (2014) |
| TaVer2-DIR3/JRL | BAA32786 | 300 | NCBI/ Yong et al. (2003)/ Song et al. (2014) |
| TaJA1-DIR4/JRL | AAR20919 | 304 | NCBI/ Song et al. (2014) |
| TaDIR1/JRL | AAC49284 | 343 | NCBI/ Ralph et al. (2007)/ Song et al. (2014)/ Kumar et al. (2018) |
| TaCDM85887-DIR/JRL | CDM85887 | 324 | NCBI |
| TaCDM85899-DIR/JRL | CDM85899 | 317 | NCBI |
| *Hordeum vulgare* | | | |
| HvJA1(DIR1/JRL) | AAA87042 | 304 | NCBI/ Lee et al. (1996); Ralph et al. (2006) |
| HvDIR2/JRL | AAA87041 | 304 | NCBI/ Lee et al. (1996); Ralph et al. (2006) |
| HvDIR3/JRL | AAB72098 | 306 | NCBI/ Lee et al. (1996); Ralph et al. (2006) |
| HvDIR/JRL | BAK00987 | 304 | NCBI |
| *Brachypodium distachyon* | | | |
| Bd39920-DIR/JRL | Bradi4g39920 | 303 | Phytozome |
| Bd39910-DIR/JRL | Bradi4g39910 | 302 | Phytozome |
| Bd05700-DIR/JRL | Bradi4g05700 | 323 | Phytozome |
| Bd21150-DIR/JRL | Bradi3g21150 | 309 | Phytozome |
| Bd24330-DIR/JRL | Bradi4g24330 | 304 | Phytozome |
| Bd48292-DIR/JRL | Bradi1g48292 | 316 | Phytozome |
| Bd01837-DIR/JRL | Bradi3g01837 | 313 | Phytozome |
| Bd48282-DIR/JRL | Bradi1g48282 | 287 | Phytozome |
| *Arabidopsis thaliana* | | | |
| At177447-JRL | AT1G73040 | 176 | NCBI |
| At19715-JRL | AT1G19715 | 601 | NCBI |

# Supplementary Figures


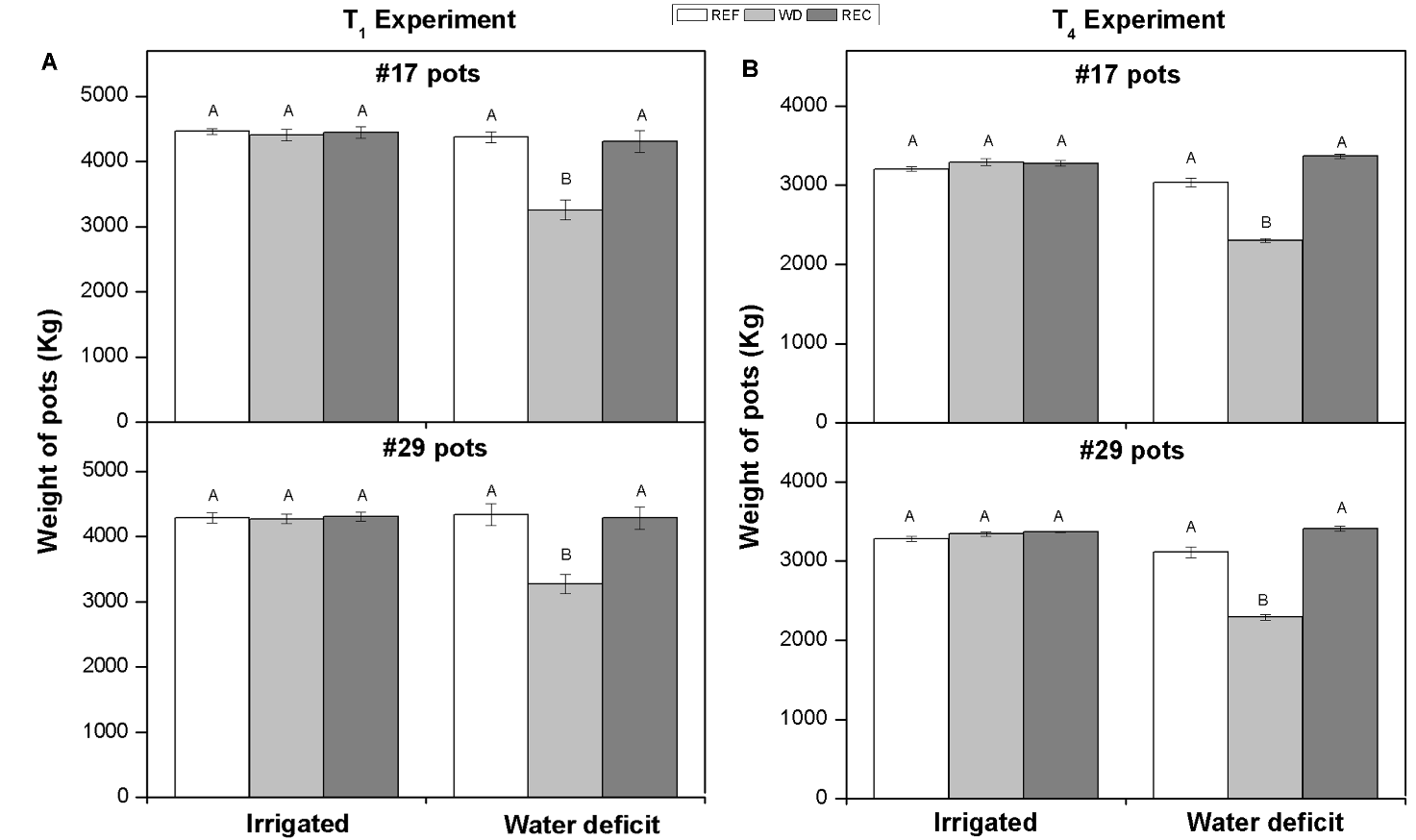


**Supplementary Figure 1.** Pot weights (Kg) were evaluated under irrigated and drought conditions in (A) T_1_ and (B) T_4_ experiments.

**
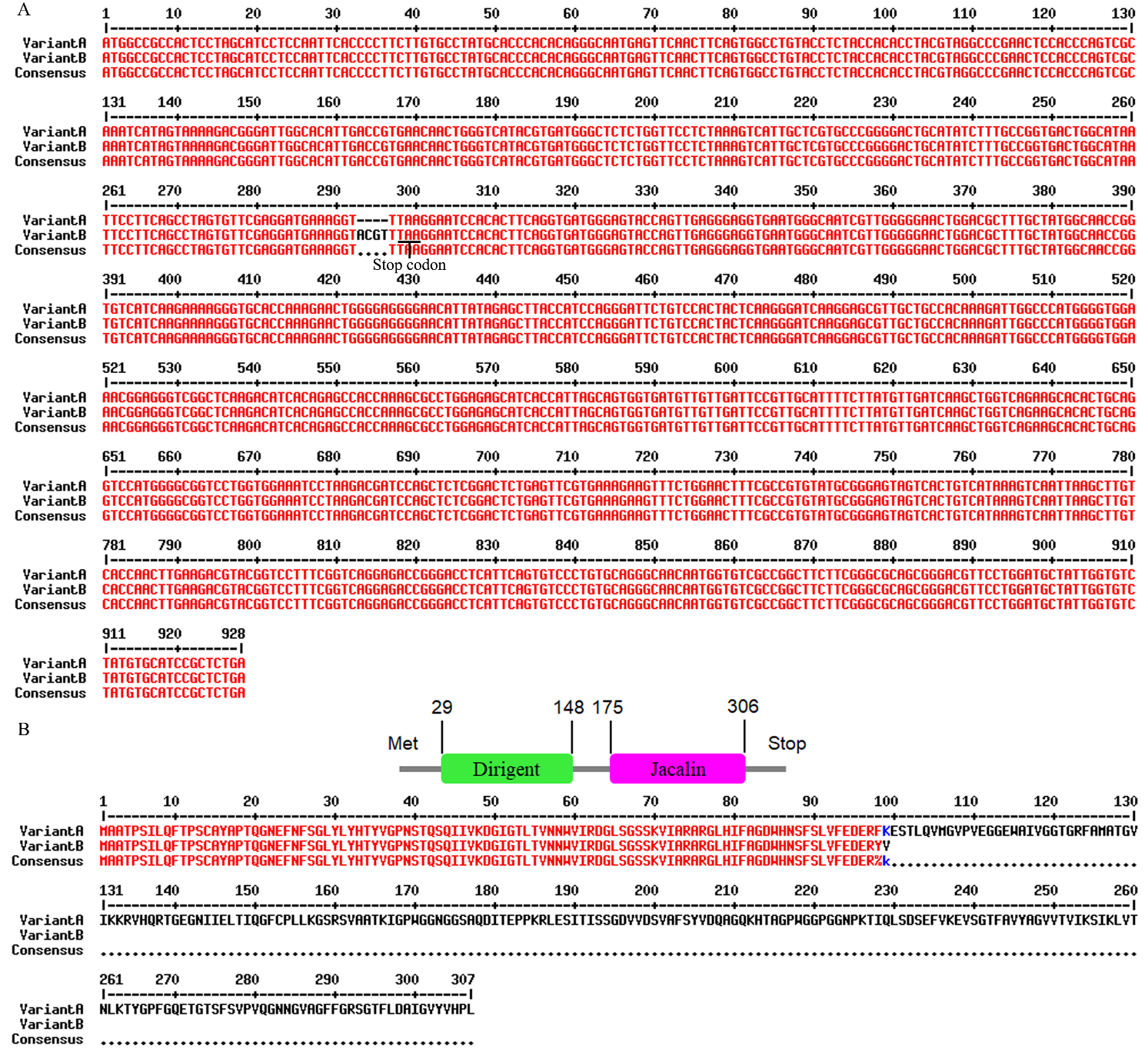
**

**Supplementary Figure 2. Allelic variants of *ShDJ* sequence.** (A) Alignment of variants showing Variant B with additional four nucleotides comparing to variant A. (B) Alignment of protein sequence provided of variants A and B. The sequences were aligned using MultAlin (<http://multalin.toulouse.inra.fr/multalin/>). Red color represent nucleotides or amino acids similarity.


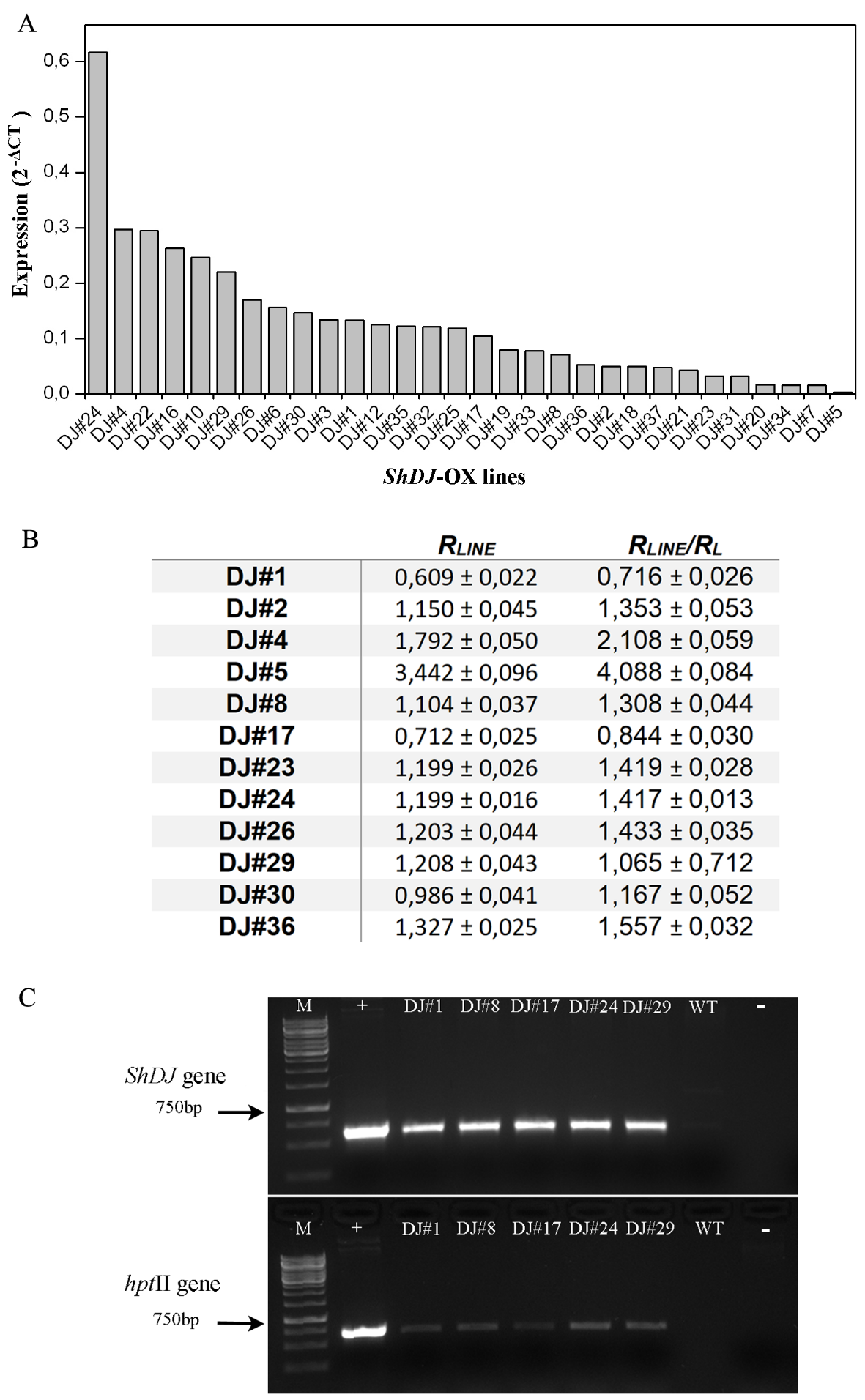


**Supplementary Figure 3. Molecular characterization of *ShDJ* lines. (A) Transcript abundance of 30 *ShDJ* lines from the T0 generation determined by RT-qPCR using primers specific for the *ShDJ* gene. Analyzes were calculated using 2^-ΔCT^ (Livak and Schmittgen, 2001) method, whereby ΔCT represents the relative quantification of a target gene and a reference gene (elF-1α). (B) Transgene copy number by TaqMan® using the *hpt*II gene as target and SPS as a control. (C) PCR product amplification of *ShDJ* (ShDJ-F and T35S-R) and *hpt*II (Hyg-F and Hyg-R) sequences in five transformed rice lines from the T1 generation. M: ladder 1 Kb. WT: wild-type plant. +/-: positive (vector) and negative controls (blank), respectively.**


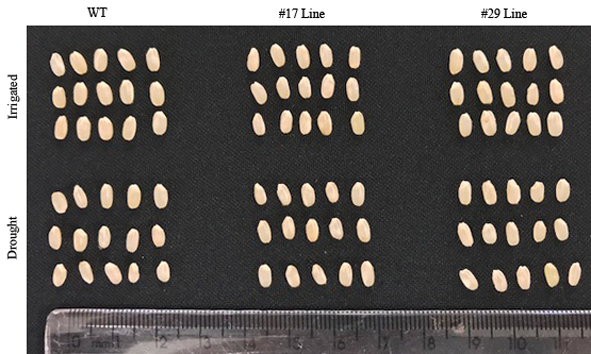


**Supplementary Figure 4. Seed size of T5 progeny under normal and drought stress conditions.**


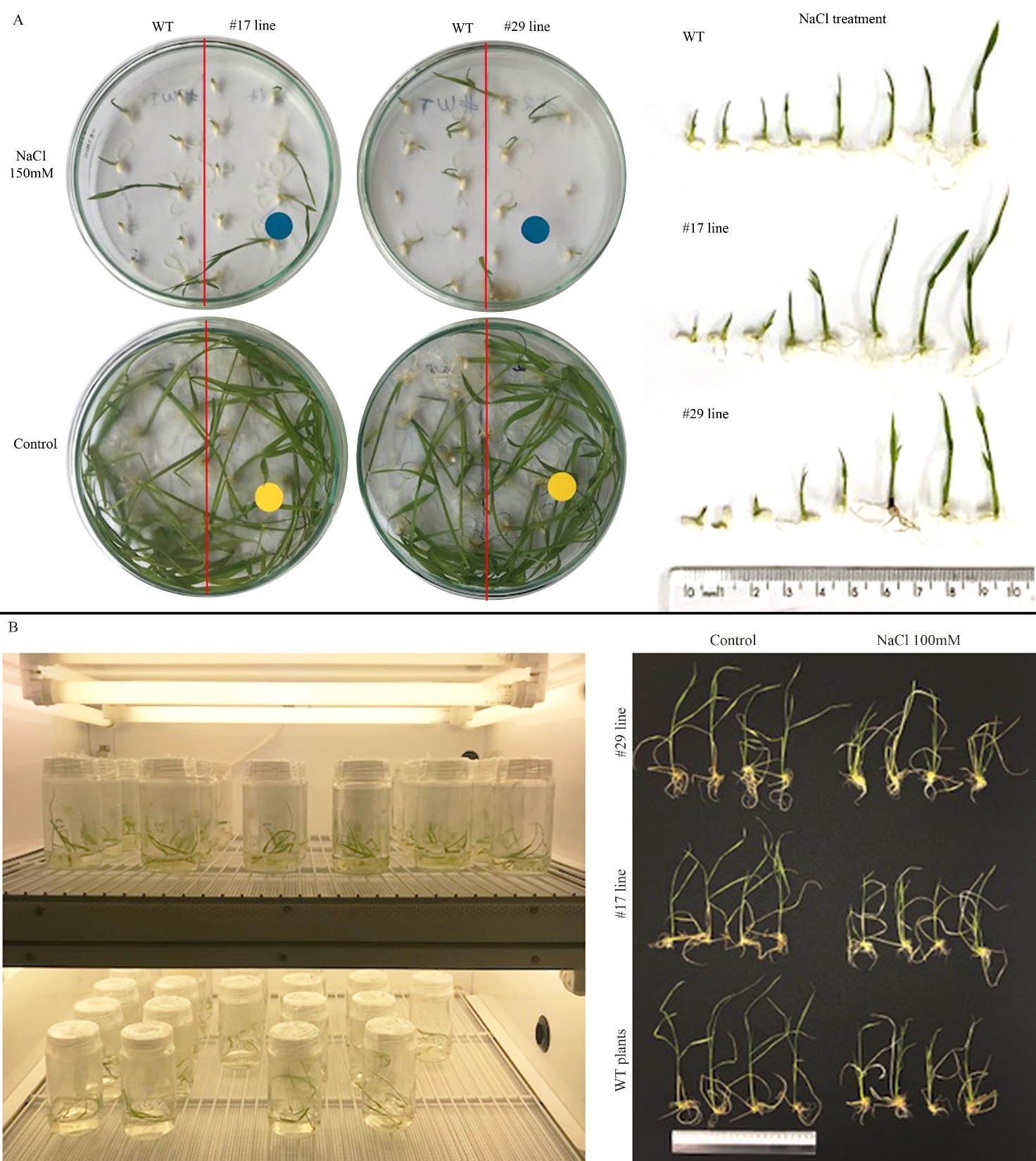


**Supplementary Figure 5****.** Salinity resistance of *ShDJ*-overexpressing plants. (A) Seeds were germinated on MS medium containing 0 or 150 mM NaCl for 10 days. (B) Seedlings maintained on MS medium containing 0 or 100 mM NaCl for 6 days and recovery for 6 days.

Salinity stress test

To assess salt tolerance, two tests were performed in transgenic lines and WT plants. Seeds of rice (*Oryza sativa*) were sterilized, and then germinated on MS medium containing 0 or 150 mM NaCl (Kumar et al., 2014) for 10 days and incubated at 27°C in a growth chamber under a 16 h/8 h (light/dark) photoperiod (Percival).

A second salt test was performed to evaluate the effects of NaCl on plant growth (Supplementary Figure 5b). Ten-days after germination, seedlings grown on MS agar medium were transferred into fresh MS liquid medium supplemented with 100 mM NaCl (He et al., 2017) and incubated at 27°C in a growth chamber under a 16 h/8 h (light/dark) photoperiod (Percival). After 6 days of NaCl treatment, transgenic lines and WT seedlings were transferred into fresh MS liquid medium for 6-days recovery.
